# Manipulation of innate immune signaling pathways by SARS-CoV-2 non-structural proteins

**DOI:** 10.3389/fmicb.2022.1027015

**Published:** 2022-11-21

**Authors:** Yongxu Lu, Hendrik A. Michel, Pei-Hui Wang, Geoffrey L. Smith

**Affiliations:** ^1^Department of Pathology, University of Cambridge, Cambridge, United Kingdom; ^2^Advanced Medical Research Institute, Cheeloo College of Medicine, Shandong University, Jinan, China

**Keywords:** SARS-CoV-2, innate immunity, Nsp6, Orf6, Orf8, IRF3, NF-κB, JAK-STAT

## Abstract

Severe acute respiratory syndrome coronavirus 2 (SARS-CoV-2), the causative agent of the current coronavirus disease 2019 (COVID-19) pandemic, induces an unbalanced immune response in the host. For instance, the production of type I interferon (IFN) and the response to it, which act as a front-line defense against virus invasion, are inhibited during SARS-CoV-2 infection. In addition, tumor necrosis factor alpha (TNF-α), a proinflammatory cytokine, is upregulated in COVID-19 patients with severe symptoms. Studies on the closely related betacoronavirus, SARS-CoV, showed that viral proteins such as Nsp1, Orf6 and nucleocapsid protein inhibit IFN-β production and responses at multiple steps. Given the conservation of these proteins between SARS-CoV and SARS-CoV-2, it is not surprising that SARS-CoV-2 deploys similar immune evasion strategies. Here, we carried out a screen to examine the role of individual SARS-CoV-2 proteins in regulating innate immune signaling, such as the activation of transcription factors IRF3 and NF-κB and the response to type I and type II IFN. In addition to established roles of SARS-CoV-2 proteins, we report that SARS-CoV-2 proteins Nsp6 and Orf8 inhibit the type I IFN response but at different stages. Orf6 blocks the translocation of STAT1 and STAT2 into the nucleus, whereas ORF8 inhibits the pathway in the nucleus after STAT1/2 translocation. SARS-CoV-2 Orf6 also suppresses IRF3 activation and TNF-α-induced NF-κB activation.

## Introduction

In the last 20 years, coronaviruses (CoVs) have caused three global outbreaks of infectious disease in humans: Severe acute respiratory syndrome (SARS, 2002, caused by SARS-CoV), Middle East respiratory syndrome (MERS, 2012, caused by MERS-CoV) and coronavirus disease 2019 (COVID-19, caused by SARS-CoV-2). SARS-CoV-2 is a member of the genus *Betacoronavirus*, subgenus *Sarbecovirus* of the family *Coronavirus*. Like other CoVs, SARS-CoV-2 is an enveloped virus with prominent surface spikes that give the virion its crown (corona)-like appearance. SARS-CoV-2 has a large, single-stranded, positive sense, RNA genome of 29.9 kb and shares 79% nucleotide identity with SARS-CoV, but only 50% identity with MERS-CoV (Lu et al., 2020). SARS-CoV-2 is most closely related to CoVs isolated from bats from China called SARS-related coronaviruses (SARSr-CoV). The closest relatives, Bat RaTG13-CoV and Bat RmYNO2, were isolated from horseshoes bats in Yunnan province, with the former sharing 96.3% nucleotide identity with SARS-CoV-2. The SARS-CoV-2 genome encodes at least 14 open reading frames (ORFs; Chan et al., 2020; Gordon et al., 2020) in addition to untranslated regions (UTRs) at the 5′ and 3′ ends. The translation products of the ORFs include polyprotein 1a (pp1a) and pp1ab, which are cleaved into non-structural proteins (Nsp)1 to Nsp16 by viral papain-like cysteine protease (PLpro and Nsp3) and main protease (Mpro and Nsp5); 9 accessory proteins Orf3a, Orf3b, Orf6, Orf7a, Orf7b, Orf8, Orf9b, Orf9c, and Orf14; and four structural proteins, spike (S), membrane (M), envelop (E), and nucleocapsid (N). A previous transcriptomic study also identified transcripts of unknown ORFs during SARS-CoV-2 infection, however the translated products from these remains to be confirmed (Kim et al., 2020).

Innate immune responses are essential to control invading pathogens such as CoVs. Host cells sense infection by pathogens *via* cellular pattern recognition receptors (PRRs), which are activated by recognition of pathogen-associated molecular patterns (PAMPs) such as viral nucleic acids. PRRs activate downstream signaling pathways that are involved in regulating innate immune responses against virus infection. Activation of the transcription factors interferon (IFN) regulatory factor 3 (IRF3) and nuclear factor-κB (NF-κB) promotes the transcription of type I IFN genes, and then the synthesis and secretion of type I IFNs (O’Neill and Bowie, 2010). Type I IFNs then bind to the type I IFN receptor at the cell surface to activate the type I IFN response *via* the JAK–STAT signaling pathway. Type I IFNs upregulate the transcription of IFN-stimulate genes (ISGs) with promoters containing the IFN-stimulated response element (ISRE) leading to translation of ISG products (Katze et al., 2002). Examples of ISG products include the IFN-induced protein with tetratricopeptide repeats (IFIT)1, IFIT2, IFIT3, and IFITM (Vladimer et al., 2014), which can restrict virus infection (Diamond and Farzan, 2013).

Activation of the transcription factor NF-κB leads to its translocation into the nucleus and the transcription of NF-κB-responsive genes. Some of the encoded proteins promote inflammation, apoptosis and further IFN-β expression, whereas others have direct antiviral activity (Thanos and Maniatis, 1995; Hayden and Ghosh, 2004). TNF-α is a potent physiological inducer of NF-κB leading to NF-κB responsive gene expression (Duh et al., 1989; Lowenthal et al., 1989; Osborn et al., 1989), and can restrict infection by influenza virus (IAV; Seo and Webster, 2002), hepatitis C virus (HCV), hepatitis E virus (HEV; Wang et al., 2016) and poxviruses (Bartee et al., 2009). However, patients with severe COVID-19 typically have high plasma levels of TNF-α (Gong et al., 2020; Huang et al., 2020), which is suggested to enhance inflammatory responses, thereby contributing to the severity of the disease (Del Valle et al., 2020).

To evade these host defences, viruses have evolved proteins that interfere with the recognition by PRRs, or the activation of innate immune signaling pathways. For example, SARS-CoV expresses several inhibitors of IRF3 and the type I IFN response. These include proteins inhibiting the sensing of viral RNA in the cytoplasm; namely Nsp3, Nsp10, Nsp14, Nsp15, Nsp16 and the structural protein N. In addition, Nsp1, Nsp3, Orf3b, Orf6, Orf9b and structural protein M interact with IRF3, MAVS, TBK1 and IKKe to suppress activation of the IRF3 pathway (Sa Ribero et al., 2020). Unbiased screens on SARS-CoV-2 proteins carried out by different groups have identified several viral inhibitors of innate immune signaling that act in similar ways (Lei et al., 2020; Xia et al., 2020). The cellular responses to type I IFNs and the pro-inflammatory cytokines produced and secreted in response to IRF3 activation, are also strongly suppressed by SARS-CoV-2 infection (Zhang et al., 2021b). For example, SARS-CoV-2 Nsp1 decreases STAT1 phosphorylation and blocks host mRNA translation, therefore also repressing the induction of ISGs, while Nsp13 and N repress phosphorylation of both STAT1 and STAT2 (Mu et al., 2020). Finally, Orf6 interferes with the nuclear translocation of the STATs by interaction with karyopherin alpha 2 (KPNA2) as well as interacting with Nup98 (Miorin et al., 2020; Xia et al., 2020).

Here, we have undertaken a screen to identify SARS-CoV-2 proteins that regulate innate immune signaling. To do this, we expressed each SARS-CoV-2 protein by transfection and used reporter gene assays to measure the activation of IRF3, NF-κB and type I IFN signaling. We also studied the translocation of transcription factors into the nucleus by immunofluorescence microscopy (IF), and quantified the level of transcription of endogenous genes by quantitative reverse transcription PCR (RT-qPCR). Consistent with other studies, we found that several SARS-CoV-2 proteins inhibited innate immune signaling and, in addition, gave new insight on proteins Nsp6, Orf6 and Orf8. Nsp6 and Orf8 were shown to inhibit type I IFN signaling without blocking STAT1 or STAT2 translocation into the nucleus. Orf8, but not Nsp6, was able to inhibit pathway activation induced by IRF9-S2C, a fusion protein composed of IRF9 and the C-terminal transactivation domain of STAT2 that constitutively activates the pathway independent of exogenous stimulation. In contrast to Nsp6 and Orf8, Orf6 blocked translocation of STAT1 or STAT2 into the nucleus following stimulation with either type I or type II IFN. Lastly, Orf6 also blocked activation of both IRF3 and NF-κB. In the latter case, Orf6 suppressed TNF-α-induced p65 translocation. In summary, this study showed that Nsp6 of SARS-CoV-2 inhibits the type I IFN response, and Orf6 suppresses TNF-α-induced NF-κB activation.

## Materials and methods

### Cell lines and plasmids

Human HEK-293 T and HeLa cells were maintained in DMEM supplemented with 50 μg/ml penicillin/streptomycin (Gibco) and 10% fetal bovine serum (FBS; Pan Biotech). Plasmids for expression of SARS-CoV-2 proteins tagged at the C terminus with a FLAG epitope tag were described previously (Zhang J. et al., 2020). The reporter gene assay plasmids containing either ISRE (pISRE-Luc) or NF-κB (pNF-κB-Luc) responsive promoters driving firefly luciferase expression were kindly provided by Professor Andrew Bowie, Trinity College, Dublin, Ireland. The ISG56.1 reporter plasmid (pISG56.1-Luc) was a gift from Dr. Ganes Sen, Cleveland Clinic, Cleveland, OH, United States. Plasmid pTK-RL that gives constitutive expression of *Renilla* luciferase was purchased from Promega. pcDNA3.TAP-C6 and pcDNA3.IRF9-S2C were described previously (Stuart et al., 2016). A plasmid expressing IRF3-5D (a constitutively active mutant of IRF3) was kindly provided by Professor John Hiscott, Lady Davis Institute for Medical Research, McGill University, and a TAP-tagged (tandem Strep and Flag epitopes) p65 expression plasmid was described elsewhere (Pallett et al., 2019).

### Antibodies and reagents

Antibodies used in this study are listed in Table 1. The plasmids were transfected using TransIT-LT1 transfection reagent (Mirus, MIR 2306). The cytokines used in the reporter assay and immunofluorescence microscopy are IFN-α (Peprotech, 300-02AA), IFN-γ (Peprotech, 300–02) and TNF-α (Peprotech, 300-01A). Sendai virus (SeV) Cantell strain (Licence No. ITIMP 17.0612A) was a kind gift from Professor Steve Goodbourn, St George’s Hospital Medical School, University of London. SeV infection was performed using 40 haemagglutination units (HAU)/mL.

**Table 1:** Antibodies used in this study.

Target	Species	Dilution used	Manufacturer	Product code
FLAG	Mouse	1:1,000 (IF)	Sigma-Aldrich	F3165
		1:1,000 (WB)		
FLAG	Rabbit	1:1,000 (IF)	Sigma-Aldrich	F7425
		1:1,000 (WB)		
STAT2	Rabbit	1:500 (IF)	Abcam	ab32367
		1:1,000 (WB)		
STAT1	Rabbit	1:500 (IF)	Abcam	ab234400
		1:500 (WB)		
Phospho-STAT1	Rabbit	1:500 (WB)	Abcam	ab30645
p65	Mouse	1:500 (IF)	Santa Cruz	sc-8,008
GAPDH	Mouse	1:1,000 (WB)	Sigma-Aldrich	G8795
α-tubulin	Mouse	1:2,000 (WB)	Santa Cruz	sc-393,512
IFITM1-3	Mouse	1:1,000 (WB)	Santa Cruz	sc-374,026
IFIT3	Mouse	1:1,000 (WB)	Santa Cruz	sc-393,512
α-Mouse-546	Donkey	1:20,000 (IF)	Invitrogen	A10036
α-Rabbit-488	Goat	1:20,000 (IF)	Invitrogen	A1108
IRDye^®^ 800CW α-Mouse IgG	Donkey	1:10,000 (WB)	LI-COR	926–32,212
IRDye^®^ 800CW α-Rabbit IgG	Goat	1:10,000 (WB)	LI-COR	926–32,211
IRDye^®^ 680RD α-Mouse IgG	Donkey	1:10,000 (WB)	LI-COR	926–68,072
IRDye^®^ 680RD α-Rabbit IgG	Goat	1:10,000 (WB)	LI-COR	926–68,071

### Reporter gene assays

HEK293 T cells were seeded in 96-well plates at a density of 3 × 10^5^ cells/well and were cultured overnight. Cells were then transfected in quadruplicate with the indicated firefly luciferase reporter plasmids (pISG56.1-Luc, pISRE-Luc or pNF-κB-Luc at 100 ng/well), a reporter plasmid expressing *Renilla* luciferase driven by the herpes simplex virus thymidine kinase gene promoter (pTK-RL, 10 ng/well) and the SARS-CoV-2 protein expression pCAG vectors (100 ng/well). Transfected cells were infected with Sendai virus (SeV) overnight to activate IRF3, or were stimulated with IFN-α (1,000 U/ml) for 8 h to activate the JAK–STAT pathway, or were stimulated with TNF-α (20 ng/ml) for 8 h to activate NF-κB. Stimulated cells were lysed with 50 μl passive lysis buffer (Promega, E1941) and kept at −20°C to conserve the bioactivity of the reporters. Firefly luciferase activity of 8 μl cell lysate was measured with 50 μl reagent (20 mM tricine, 2.67 mM MgSO_4_·7H_2_O, 0.1 mM EDTA, 33.3 mM DTT, 530 μM ATP, 270 μM acetyl-CoA, 132 μg/ml luciferin (Prolume, 306–500), 5 mM NaOH, 0.26 mM MgCO_3_Mg(OH)_2_·5H_2_O). *Renilla* luciferase activity served as an internal transfection control and was measured with 50 μl reagent of 2 μg/ml coelenterazine (Nanolight Technology, 350–10) in PBS. Both firefly and *Renilla* luciferase luminescence values were measured with a FLUOstar luminometer (BMG Labtech). Relative promoter activity was measured by determining the firefly/*Renilla* luminescence ratios, and the fold inductions were calculated relative to the respective unstimulated controls. Aliquots of the lysates were collected and analyzed by immunoblotting to confirm SARS-CoV-2 protein expression.

### Immunofluorescence

HeLa cells were seeded on 19-mm glass coverslips in 6-well plate at 10^5^ cells/well. Cells were transfected overnight with 1 μg of each pCAG vector expressing a SARS-CoV-2 protein using polyethylenimine (PEI) at a ratio of 1:3 of DNA (μg): PEI (μl). Transfected cells were then stimulated for 30 min with IFN-α, IFN-γ or TNF-α to activate type I IFN, type II IFN responses or NF-κB pathway, respectively. The cells were then washed twice with PBS and fixed in 4% formaldehyde, 0.02 g sucrose/ml in PBS at room temperature (RT) for 10 min. The coverslips were washed twice with PBS and cells were permeabilised in 0.5% NP40 and 0.1 g/ml sucrose in PBS at RT for 10 min. Then the coverslips were washed twice with 1% FBS/PBS. Cells were stained with primary antibody for 2 h by incubation in 1% FBS/PBS, washed with 1% FBS/PBS twice and incubated with the secondary antibody for 1 h before final washing. Primary and secondary antibodies were used at different dilutions and listed in Table 1. Coverslips were mounted with Mowiol medium (Calbiochem, 475,904) containing 0.5 μg/ml 4′, 6-diamidino-2-14 phenylindole (DAPI; Sigma Aldrich, D8417). Images of the coverslips were scanned and recorded with LSM 700 confocal microscope (Zeiss) using ZEN 16 system (Zeiss).

### Co-precipitation assay

HEK-293 T cells were co-transfected with 1.5 μg of pcDNA3.TAP-p65 and pcDNA3.HA-IκBα or pCAG.Orf6-FLAG overnight. The following day, the transfected cells were starved with DMEM without serum for 3 h, and then stimulated with TNF-α (20 ng/ml) for 1 h. The cells were then washed twice with pre-cold PBS and lysed with cell lysis buffer (PBS with 0.5% NP-40) supplemented with protease inhibitor (Merck, 11,836,153,001). The lysates were cleared by centrifugation (15,000 rpm, 5 min, 4°C) and TAP-tagged p65 was pulled down by Strep-Tactin resin (IBA, 2-5010-002). Pull-down samples were washed 3 times in lysis buffer and bound proteins were eluted by boiling for 10 min in protein buffer (50 mM Tris–HCl (pH 6.8), 2% SDS (*w*/*v*), 10% glycerol (*w*/*v*), 0.1% bromophenol blue (*w*/*v*), 100 mM BME). Samples were then analyzed by SDS-PAGE and immunoblotting with the indicated antibodies.

### Immunoblotting

Cell lysates collected from the reporter gene assay were mixed with NuPAGE LDS sample buffer (Invitrogen, NP0007) at a ratio of 3:1 and then heated at 70°C for 10 min. HEK-293 T cells were transfected with SARS-CoV-2 protein expression vectors overnight and then stimulated with IFN-α (as above) or left unstimulated. Cells were lysed in lysis buffer (50 mM Tris–HCl pH 8.0, 150 mM NaCl, 1 mM EDTA, 10% (*v*/*v*) glycerol, 1% (*v*/*v*) Triton X-100 and 0.05% (*v*/*v*) Nonidet P-40 (NP-40), protease inhibitors (Roche, 11836170001) and phosphatase inhibitors (Roche, 04906837001)). Lysates were clarified by centrifugation at 17,000 × *g* for 15 min at 4°C, and then were mixed with loading buffer (150 mM Tris–HCl, 6% SDS, 30% glycerol, 0.3% bromophenol blue, 300 mM β-mercaptoethanol) at a ratio of 2:1 and heated at 95°C for 10 min. The equivalent amounts of samples were loaded onto NuPAGE 4–12% Bis-Tris precast gels (Invitrogen, NP0336BOX) alongside protein molecular mass markers (Abcam, ab116028). Proteins separated by NuPAGE precast gels were transferred onto nitrocellulose membranes (GE Healthcare, 10600001) by Trans-Blot Turbo transfer system (Bio-Rad, 1,704,150) in transfer buffer (25 mM Tris·HCl, 250 mM glycine, and 20% methanol). The transferred membranes were dried at RT and blocked with 5% skimmed milk in PBS with 0.1% tween (PBST) for 30 min at RT with constant agitation. The membranes were incubated with primary antibody overnight at 4°C with constant agitation, and then were washed three times with PBST before incubation with secondary antibody for 2 h. Then the membranes were washed three times with PBST, dried at RT, and scanned by the LI-COR Odyssey imaging system.

### Reverse transcription and quantitative PCR

HEK-293 T cells seeded in 12-well plates (8 × 10^5^ cells/well) were transfected with plasmids expressing Orf6, Orf8 or empty vector (EV) in triplicates. Transfected cells were starved for 3 h by incubation with medium without serum and then were mock-stimulated or stimulated with TNF-α (20 ng/ml) for 1 or 6 h. Total RNA from the cells was extracted by RNeasy Mini Kit (Qiagen, 74,106) and then reverse transcribed into complementary DNA (cDNA) with oligo-dT primers (Thermo Scientific, SO132) and SuperScript III reverse transcriptase kit (Invitrogen, 18,080,085). The level of mRNA of NF-κB-responsive genes *NFKBIA*, *CCL2* and *CXCL10* and internal control gene *GAPDH* were quantified by quantitative PCR (qPCR) using fast SYBR green master mix (Applied Biosystems, 4385612) and ViiA 7 real-time PCR system (Life Technologies). The gene specific primers used in the qPCR were from MERCK. Details of the primers are listed in Supplementary Table S1.

### Statistical analysis

Statistical analysis was performed using Unpaired Student’s t tests, carried out with the statistics module of GraphPad PRISM 7. Statistical significance is expressed as follows: not significant (ns), ^*^p < 0.05, ^**^p < 0.01, ^***^p < 0.001, ^****^p < 0.0001.

## Results

### SARS-CoV-2 proteins interfere with IRF3 activation, the type I IFN response and the TNF-α-induced NF-κB pathway

**Figure 1:**
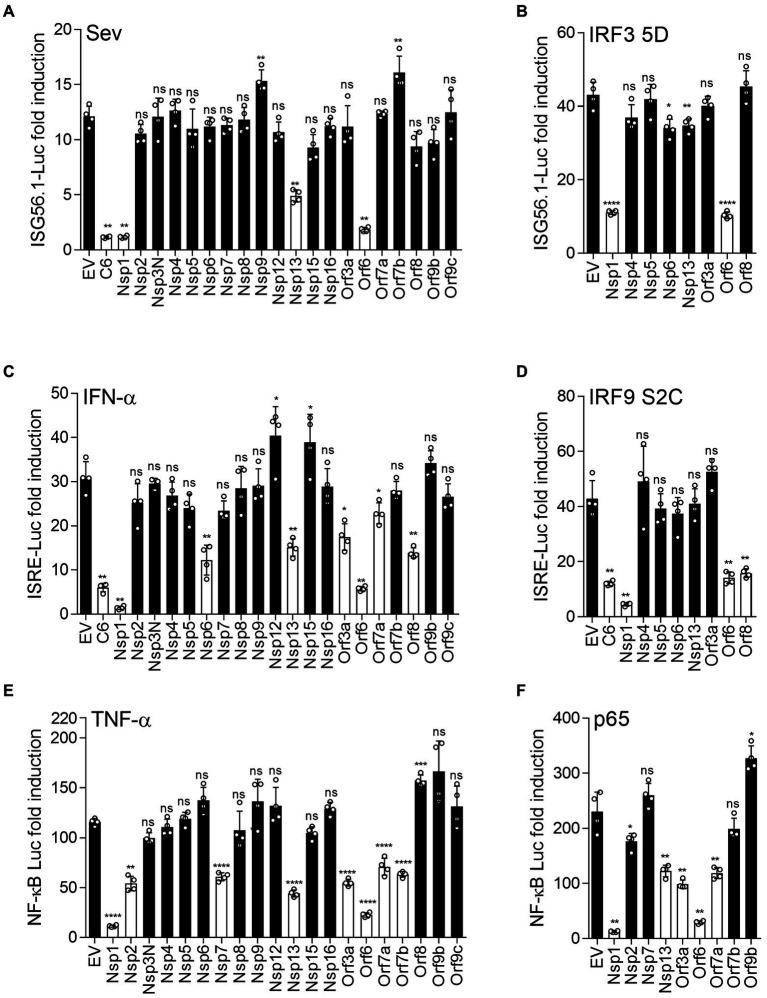
SARS-CoV-2 proteins inhibit innate immune pathway activation. HEK293 T cells were transfected with reporter plasmids ISG56.1-firefly luciferase (Fluc) **(A,B)**, ISRE-Fluc **(C,D)**, or NF-κB-Fluc **(E,F)**, TK-*Renilla*, and plasmids expressing the indicated SARS-CoV-2 proteins fused to a FLAG epitope at the C terminus. After 18 h, transfected cells were stimulated by infection with 40 HAU/ml SeV **(A)**, 1,000 unit/ml IFN-α **(C)**, or 40 ng/ml TNF-α **(E)**. Cells in **(B)**, **(D)**, or **(F)** were transfected simultaneously with reporter plasmids and plasmids expressing IRF3-5D, IRF9-S2C or p65 to activate the IRF3 pathway, IRF9/STAT2-mediated ISG expression, or the NF-κB pathway, respectively. After stimulation (overnight infection with SeV, or 6 h with IFN-α or TNF-α), cell lysates were harvested, and firefly luciferase activity was measured and normalized to *Renilla* luciferase control. The fold induction of firefly luciferase is shown relative to unstimulated controls. Data shown are from 4 technical replicates and are representative of three independent experiments. Error bars represent standard deviation. The *p*-values were calculated using the unpaired Student’s t-test relative to empty vector. (^*^p < 0.05, ^**^p < 0.01, ^***^p < 0.001, ^****^p < 0.0001).

To investigate inhibition of innate immune signaling by SARS-CoV-2 proteins, we used reporter gene assays to screen each SARS-CoV-2 protein for the ability to inhibit activation of IRF3, NF-κB or the JAK–STAT pathway downstream of type I IFN. To do this, reporter plasmids pISG56.1-Luc, pNF-κB-Luc or pISRE-Luc were transfected into HEK-293 T cells together with a plasmid expressing each individual SARS-CoV-2 protein. A day later the pathways were activated by infection with SeV (for IRF3; Figure 1A), or addition of IFN-α (for ISRE; Figure 1C) or TNF-α (for NF-κB; Figure 1E) and the luciferase activity was measured compared to empty vector (EV) control. The viral proteins assessed were Nsp1, Nsp2, Nsp3N, Nsp4-9, Nsp12, Nsp13, Nsp15, Nsp16, Orf3a, Orf6, Orf7a, Orf7b, Orf8, Orf9b and Orf9c and immunoblotting showed expression of these proteins, albeit at slightly different levels (Supplementary Figure S1). Other SARS-CoV-2 non-structural proteins (Nsp3C, Nsp10, Nsp11 and Nsp14) were not included in this study as they failed to be expressed adequately in HEK-293T cells. Vaccinia virus (VACV) protein C6, an inhibitor of IRF3 activation and type I IFN-induced JAK–STAT signaling, was included as a positive control (Unterholzner et al., 2011; Stuart et al., 2016; Lu et al., 2019).

Expression of Nsp1, Nsp13 and Orf6 significantly suppressed the IRF3-dependent ISG56.1-driven luciferase expression, whereas the other proteins were not inhibitory, except for the positive control VACV protein C6 (Figure 1A). Two prior studies reported that Nsp1, Nsp13 and Orf6 inhibited activation of the IFN-β promoter induced by SeV infection (Lei et al., 2020; Yuen et al., 2020). However, since the IFN-β promoter has three cis-acting elements that are bound by transcription factors ATF-2/c-Jun, IRF3/IRF7 and NF-κB (Wathelet et al., 1998), it was not evident which of these were inhibited by Nsp1, Nsp13 and Orf6. Data in Figure 1A show that Nsp1, Nsp13 and Orf6 inhibit IRF3 activation. To map at which stage in the IRF3 pathway these inhibitors were acting, ISG56.1 reporter expression was induced by expression of a constitutively active form of IRF3 (IRF3-5D; Lin et al., 1998). This activation was suppressed by Nsp1 and Orf6 (Figure 1B), indicating these proteins inhibit the pathway downstream of IRF3 phosphorylation. In contrast, Nsp13 was not inhibitory, indicating it acts upstream of IRF3 phosphorylation.

To investigate how individual SARS-CoV-2 proteins manipulate the response to type I IFN, IFN-α-induced reporter expression was measured in their presence. In this assay, ISRE-dependent firefly luciferase expression was suppressed by Nsp1, Nsp6, Nsp13, Orf3a, Orf6, Orf8, and VACV protein C6 (Figure 1C). Orf7a showed marginal inhibition in this experiment, but this was not seen consistently in repeat experiments. To map at which stage these viral proteins suppress the response to type I IFN, the expression of ISRE-driven firefly luciferase was induced by IRF9-S2C, a constitutively active transcriptional activator that activates the ISRE promoter in the absence of IFN (Kraus et al., 2003). Nsp1, Orf6, Orf8 and VACV C6 suppressed this activation (Figure 1D), indicating these viral proteins inhibit type I IFN-induced JAK–STAT signaling within the nucleus downstream of IFN-stimulated gene factor 3 (ISGF3) activation.

Next, we assessed TNF-α-mediated NF-κB activation in the presence of the SARS-CoV-2 proteins. This showed that NF-κB reporter expression was reduced in the presence of Nsp1, Nsp2, Nsp7, Nsp13, Orf3a, Orf6, Orf7a and Orf7b, but not other SARS-CoV-2 proteins (Figure 1E). To investigate this inhibition further, the NF-κB pathway was activated by ectopic expression of p65, a subunit of the NF-κB heterodimer. Activation by p65 was still inhibited by Nsp1, Nsp13, Orf3a, Orf6 and Orf7a (Figure 1F), indicating these viral proteins inhibit at or downstream of p65, whereas Nsp7 and Orf7b act upstream.

### SARS-CoV-2 proteins suppress the translocation of STAT2 and STAT1

**Figure 2:**
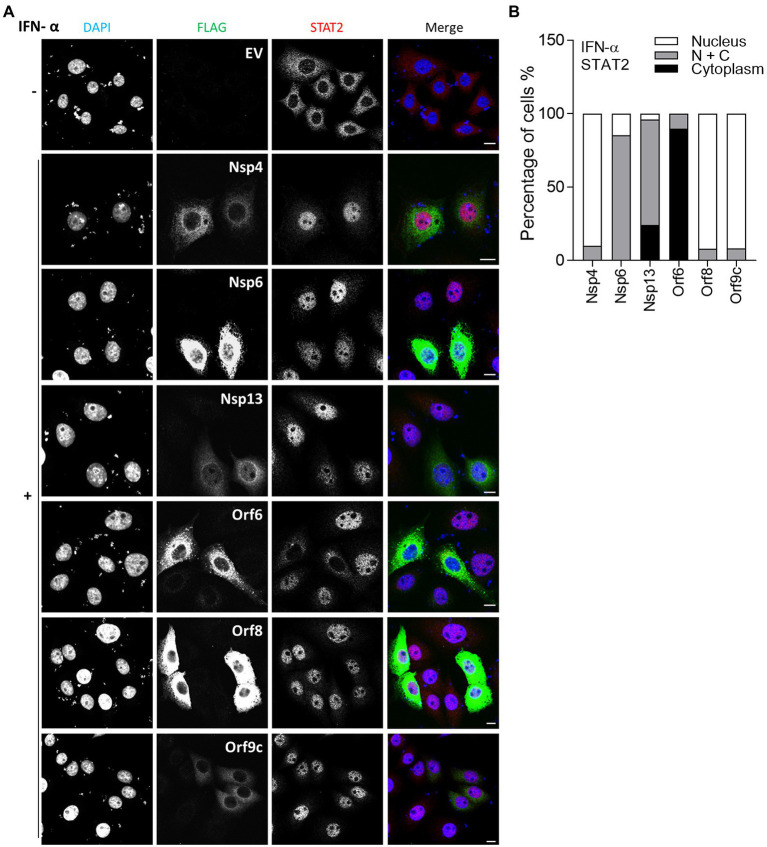
Immunofluorescence microscopy (IF) analysis of the effect of SARS-CoV-2 proteins on IFN-α induced STAT2 relocalisation. **(A)** HeLa cells were transfected with plasmids expressing the indicated SARS-CoV-2 proteins. After 12 h, transfected cells were stimulated with 1,000 unit/ml IFN-α for 1 h. The cells were then fixed, permeabilised and stained for STAT2, FLAG-tagged viral proteins and DNA with DAPI. The images are representative of the cell population. Scale bar, 10 μm. **(B)** Quantification of IFN-α-induced redistribution of STAT2 in viral proteins expression cells (*n* ≥ 20 in each condition). N + C represents staining within both the nucleus and cytoplasm.

Some viral inhibitors of innate immune signaling pathways function by blocking the translocation of transcriptional activators into the nucleus. For example, SARS-CoV Orf6 blocks the nuclear transport of STAT1 in response to IFN stimulation, by interacting with canonical import factors karyopherin alpha 2 (KPNA2) and karyopherin beta 1 (KPNB1; Frieman et al., 2007). To investigate if other SARS-CoV-2 proteins inhibited the type I IFN response in a similar way, the translocation of STAT2 into the nucleus was measured by immunofluorescence (IF) in the presence of the SARS-CoV-2 proteins after IFN-α stimulation. Representative images are shown in Figure 2A and the proportion of cells showing nuclear STAT2 following stimulation with IFN-α is shown in Figure 2B. Consistent with a recent study (Miorin et al., 2020), Orf6 blocked translocation of STAT2 induced by IFN-α (Figure 2). In our study, Nsp6 and Nsp13 also partially suppressed the translocation of STAT2, suggesting that Nsp6 and Nsp13 may inhibit the type I IFN pathway in the cytoplasm.

**Figure 3:**
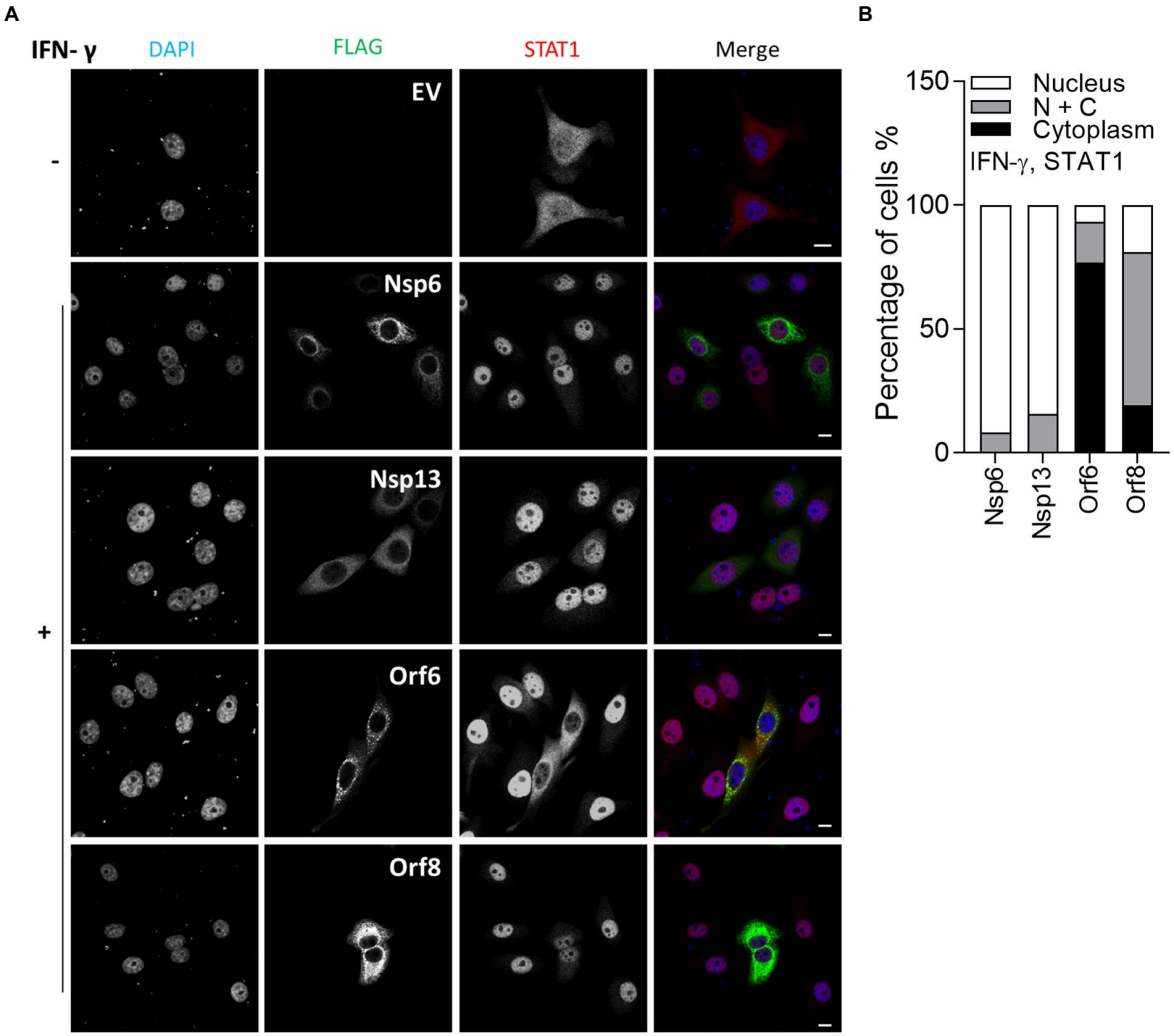
Immunofluorescence microscopy (IF) analysis of the effect of SARS-CoV-2 viral proteins on IFN-γ-induced STAT1 relocalisation. **(A)** As in Figure 2, except that transfected HeLa cells were stimulated with 250 ng/ml IFN-γ for 1 h and then stained for STAT1 rather than STAT2. Scale bar, 10 μm. **(B)** Quantification of IFN-γ-induced STAT1 redistribution (*n* ≥ 24 in each condition). N + C represents staining within both the nucleus and cytoplasm.

*In vitro* studies showed that SARS-CoV-2 infection is sensitive to type II IFN pre-treatment (Miorin et al., 2020), prompting an investigation into the inhibitory potential of viral proteins in the transfection system. Given that SARS-CoV-2 Orf6 blocks translocation of STAT2, it may also interfere with the IFN-γ-induced translocation of STAT1. To test this, IF analysis was performed as in Figure 2, except that transfected HeLa cells were stimulated with IFN-γ and then stained with antibody against endogenous STAT1. Consistent with a recent study, IFN-γ-induced STAT1 translocation was blocked by Orf6 (Miyamoto et al., 2022), and was suppressed by Orf8 (Figure 3), indicating that Orf6 and Orf8 may also inhibit the type II IFN response.

### SARS-CoV-2 Nsp6 and Orf8 suppress type I IFN-induced ISG expression

**Figure 4:**
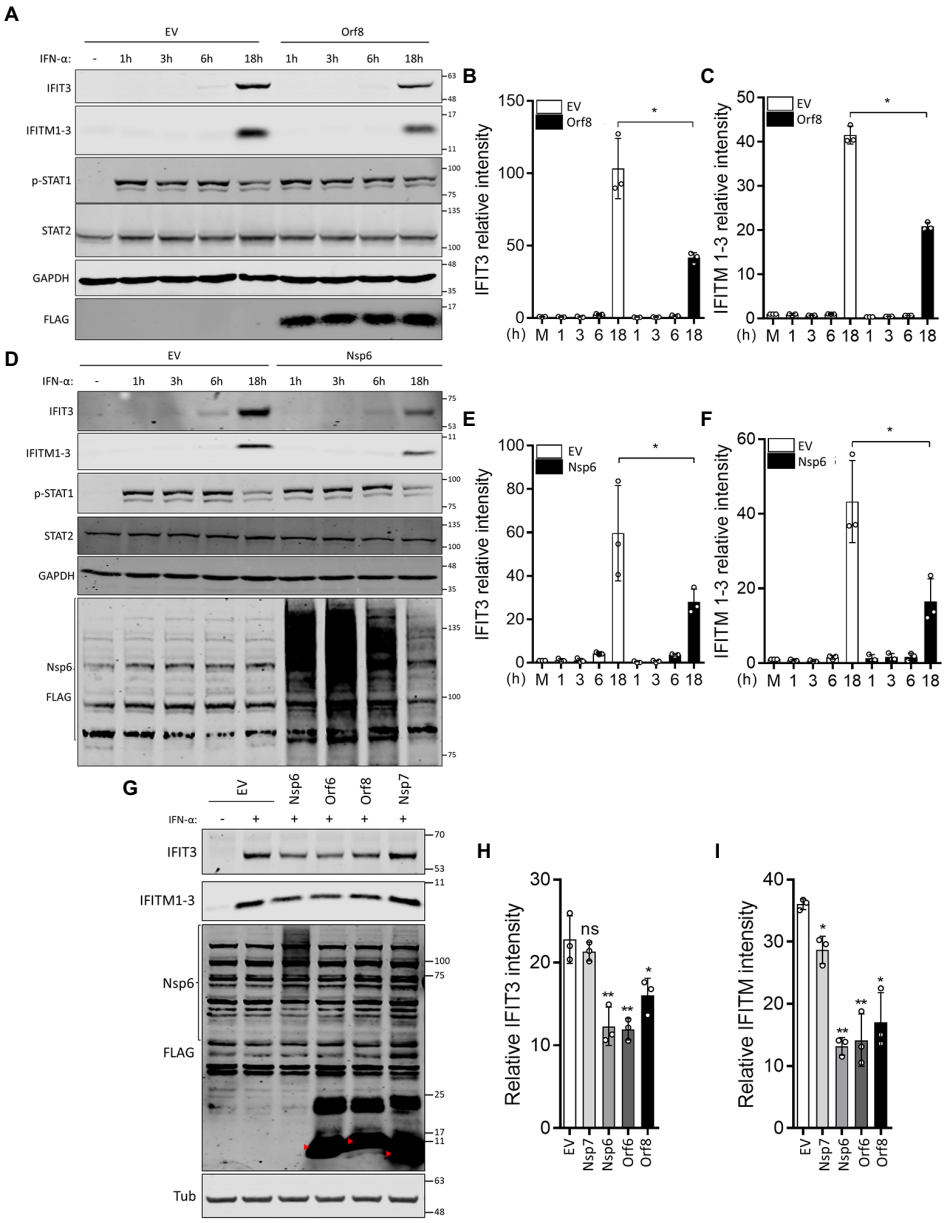
SARS-CoV-2 Orf8 and Nsp6 suppress IFN-α-induced ISG expression. **(A)** Immunoblots showing the levels of IFIT3, IFITM1-3, STAT2 and p-STAT1 expression at different times after IFN-α treatment in the presence or absence of Orf8. **(B,C)** Quantification of band intensities for IFIT3 **(B)** and IFITM1-3 **(C)** from 3 separate experiments. **(D)** as for **(A)** except that Nsp6 was expressed rather than Orf8. **(E,F)** Quantification of band intensities for IFIT3 **(E)** and IFITM1-3 **(F)** from three separate experiments. **(G)** Immunoblots showing the levels of IFIT3 and IFITM1-3 18 h after IFN-α treatment in the presence or absence of the indicated viral proteins or empty vector (EV). **(H,I)** Quantification of band intensities for IFIT3 **(H)** and IFITM1-3 **(I)** from 3 separate experiments. GAPDH **(A,D)** or tubulin **(G)** were used as loading controls. Error bars show standard deviation. The *p*-values were relative to EV. (^*^p < 0.05 and ^**^p < 0.01). For immunoblots, the positions of molecular mass markers in kDa are indicated on the right and the positions of the SARS-CoV-2 proteins are indicated by red arrowheads.

ISRE reporter gene assays performed in this study and by others suggested that Nsp6 and Orf8 inhibit the response to type I IFN (Lei et al., 2020; Xia et al., 2020), and IF analysis of STAT2 and STAT1 translocation induced by IFN-α and IFN-γ, respectively, indicated Nsp6 suppresses type I IFN-induced STAT2 translocation. To support these findings, IFN-α-induced ISG expression was analyzed in the presence of Nsp6 or Orf8. HEK-293 T cells were transfected with EV or plasmids encoding FLAG-tagged Nsp6 or Orf8 and then the transfected cells were stimulated with IFN-α. Consistent with the reporter gene assay and STAT2 translocation analysis, both Nsp6 and Orf8 significantly reduced the IFN-α-stimulated expression of IFIT3 and IFITM1-3 (Figures 4A–F). Immunoblotting of STAT2 and phospho(p)-STAT1 indicated that the levels of these proteins were not affected by Nsp6 or Orf8 (Figures 4A,D). A similar experiment was performed with other SARS-CoV-2 proteins, which acted as positive (Orf6) and negative (Nsp7) controls (Figures 4G–I) and this showed that Nsp6, Orf6 and Orf8, but not EV and Nsp7, suppressed the IFN-α-induced expression of IFIT3 and IFITM1-3 18 h after stimulation. These observations are consistent with the ISRE reporter assays (Figures 1A,B) and the IF analysis of STAT2 translocation (Figure 2).

### SARS-CoV-2 Orf6 suppresses TNF-α-stimulated NF-κB activation by retaining p65 in the cytoplasm

**Figure 5:**
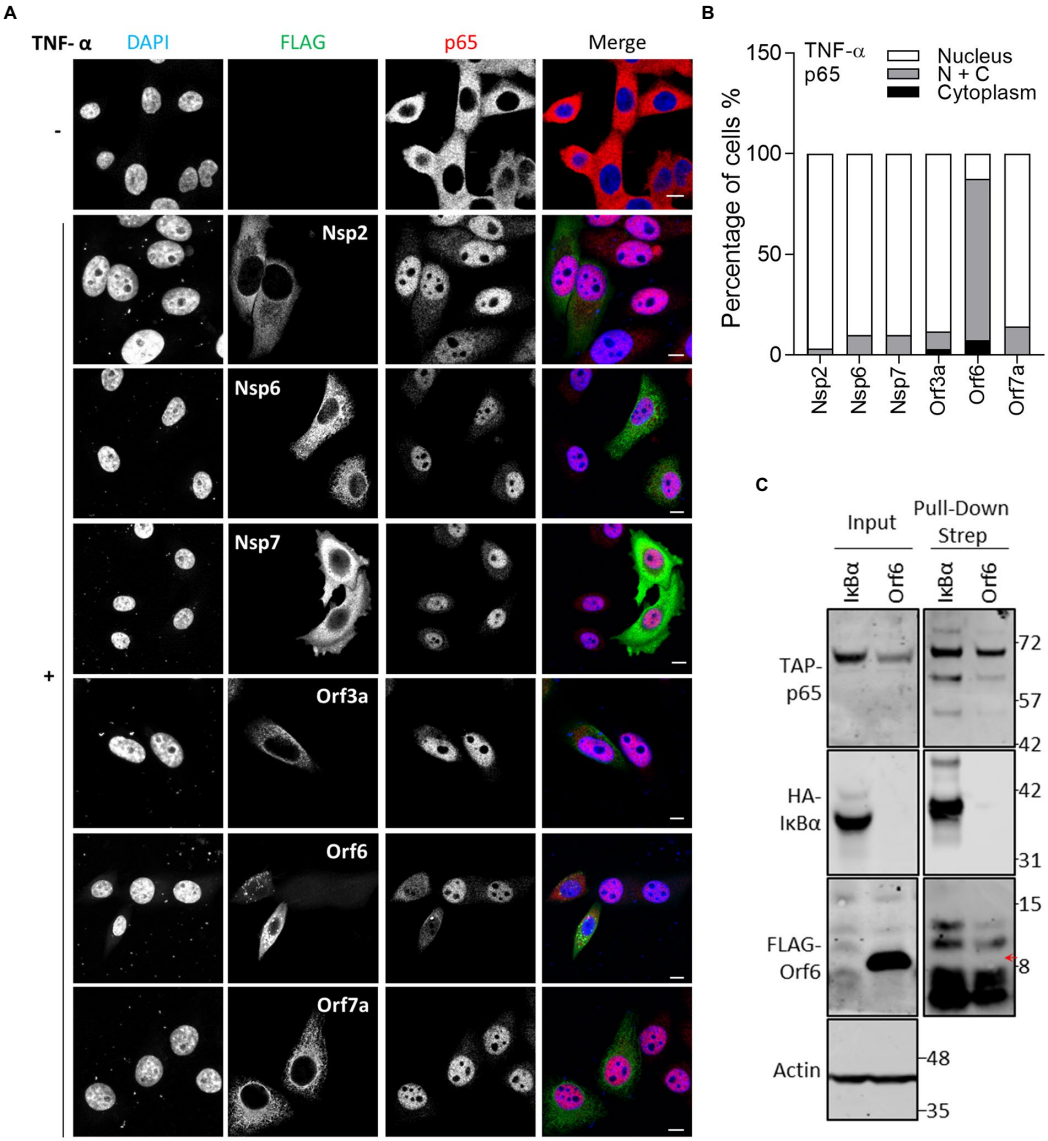
Immunofluorescence microscopy (IF) analysis of the effect of SARS-CoV-2 viral proteins on TNF-α-induced p65 relocalisation. **(A)** As in Figure 2, except that transfected HeLa cells were stimulated with 40 ng/ml TNF-α for 30 min and then stained for p65. Scale bar, 10 μm. **(B)** Quantification of TNF-α-induced p65 translocation (*n* ≥ 21 in each condition). N + C represents staining within both the nucleus and cytoplasm. **(C)** HEK-293 T cells were co-transfected with plasmids encoding TAP-tagged-p65 and HA-tagged-IκBα or FLAG-tagged Orf6. The following day, transfected cells were starved with medium without serum for 3 h, and then stimulated with TNF-α (20 ng/ml) for 1 h. The stimulated cells were harvested and lysed for pull-down *via* the TAP-epitope. Inputs (Left) and pull-down samples (Right) were analyzed by immunoblotting with the indicated antibodies. The position of the FLAG-tagged Orf6 protein is indicated by a red arrow.

The NF-κB reporter gene assay (Figure 1E) indicated that Nsp1, Nsp2, Nsp7, Nsp13 and Orf3a, Orf6, Orf7a and Orf7b negatively regulate NF-κB activation. Further analysis indicated that Nsp1, Nsp13, Orf3a, Orf5 and Orf7a could also inhibit NF-κB activation induced by p65 expression suggesting that they act at or downstream of p65 translocation. To address this possibility, the effect of these proteins on TNF-α-stimulated p65 translocation was measured by IF microscopy. This showed that Orf6, but not Nsp2, Nsp6, Nsp7, Orf3a, and Orf7a, suppressed the translocation of p65 (Figure 5). The suppression of p65 translocation suggests that Orf6 might interact with p65. To examine this, TAP-tagged p65 was expressed ectopically and then pulled down *via* its TAP-tag and its interaction partners were analyzed by immunoblotting. The known interaction of p65 with IκBα served as a positive control (Beg et al., 1992). TAP-tagged p65 co-precipitated with HA-tagged IκBα, but not FLAG-tagged Orf6 (Figure 5C). Therefore, the inhibition of NF-κB and p65 translocation is not mediated by Orf6 and p65 interaction.

**Figure 6:**
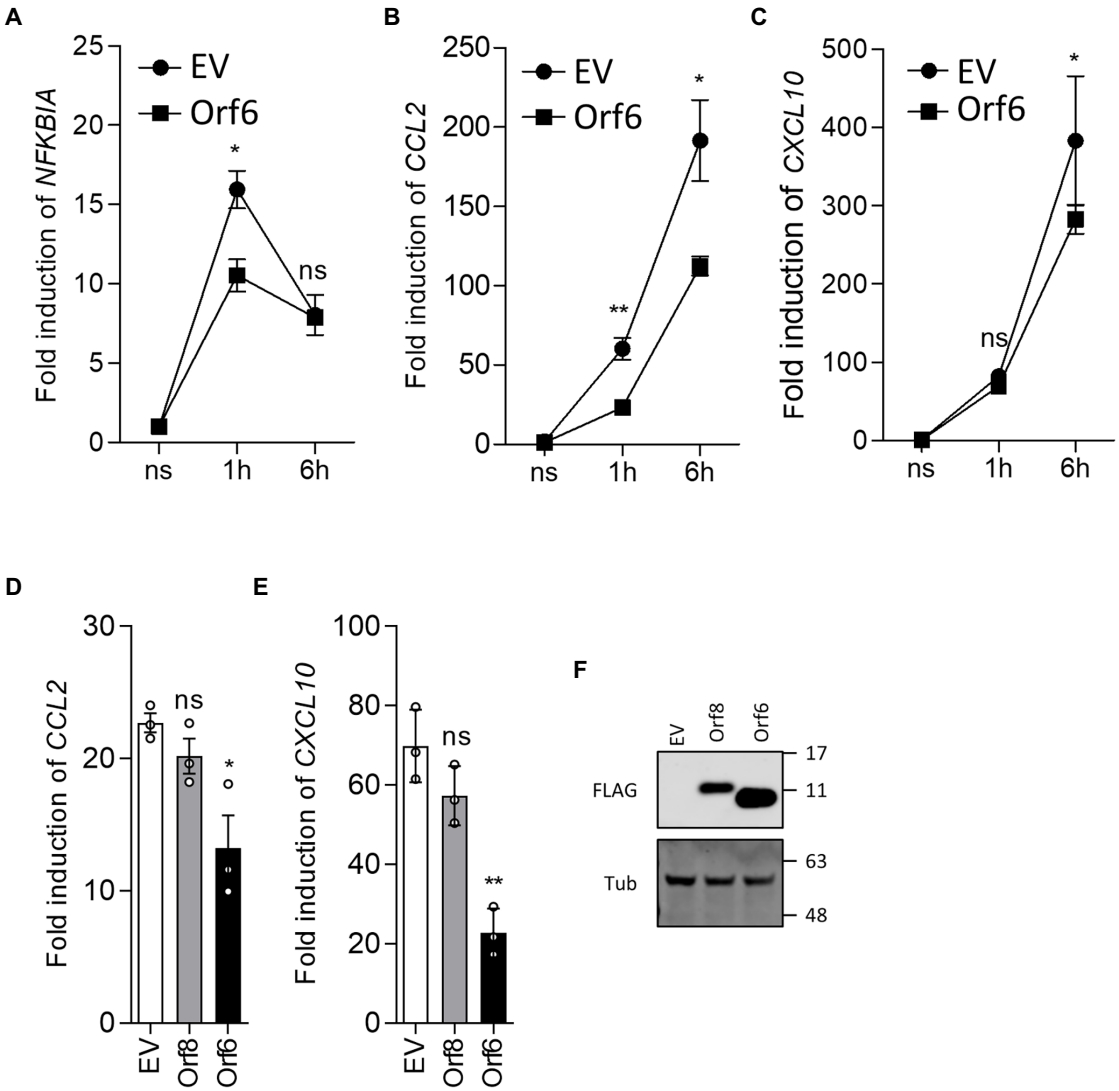
SARS-CoV-2 Orf6 suppresses the expression of NF-κB responsive genes. **(A-C)** HEK293 T cells (8 × 10^5^ cells per well) were transfected in triplicate with an empty vector (EV) or a plasmid expressing FLAG-tagged ORF6. After 24 h, the cells were starved in medium without serum for 3 h and then were stimulated with TNF-α (20  ng/ml). Stimulated cells were collected at 1 h and 6 h post stimulation and total RNA was extracted. The levels of mRNA for the NF-κB responsive genes NFKBIA, CCL2 and CXCL10 were analyzed by RT-qPCR. Figures shown are representative data from two independent experiments. **(D,E)** HEK293 T cells were transfected with plasmids expressing FLAG-tagged Orf8 or Orf6 and stimulated as in **(A-C)**. Stimulated cells were collected at 6 h post stimulation and the levels of CCL2 and CXCL10 mRNA were analyzed by RT-qPCR. Error bars show standard deviation. The p-values were relative to empty vector in **(A-C)**, or to Orf8 in **(D,E)** (^**^p < 0.01 and ^*^p < 0.05). **(F)** Immunoblot showing the levels of FLAG-tagged Orf6 and Orf8 in the transfected HEK293 T cells. For immunoblots, the positions of molecular mass markers in kDa are indicated on the right.

Inhibition of NF-κB activation was explored further by reverse transcription-quantitative PCR (RT-qPCR) analysis of endogenous NF-κB responsive genes following stimulation by TNF-α. HEK-293 T cells were transfected with either EV or the plasmid expressing Orf6 and a day later the cells were stimulated with TNF-α for 1 or 6 h. Then the mRNA level of NFKBIA, CXCL8 and CCL2 were analyzed. This showed that induction of NFKBIA, CXCL8 and CCL2 mRNA levels was inhibited by ectopic expression of Orf6 (Figures 6A–C). A repeat experiment (Figures 6D–F) also showed that Orf6, but not Orf8, suppressed CXCL8 and CCL2 transcription at 6 h post stimulation with TNF-α. Taken together these results indicate that SARS-CoV-2 Orf6 inhibits the TNF-α induced NF-κB activation by suppressing p65 translocation into the nucleus.

## Discussion

Type I IFNs are critical for controlling SARS-CoV and MERS-CoV infection (de Wilde et al., 2013; Channappanavar et al., 2019), and the accumulated evidence from clinical studies suggested that they are also essential for protecting patients from severe disease caused by SARS-CoV-2 infection (Bastard et al., 2020; Zhang Q. et al., 2020). In this study, we performed reporter gene assays in combination with IF and immunoblotting to study how SARS-CoV-2 Nsps regulate innate immune pathways that promote the production of type I IFN and the response to it. The TNF-α-activation of NF-κB, another essential innate immune pathway in controlling pathogen invasion, was also evaluated in the presence of the SARS-CoV-2 proteins by reporter gene assay, IF and RT-qPCR. The initial reporter gene assay screening revealed that: (1) Nsp1, Nsp13 and Orf6 suppressed SeV-activation of the IRF3 pathway; (2) Nsp1, Nsp6, Nsp13, Orf3a, Orf6 and Orf8 suppressed IFN-α-induced JAK-STA signaling; and (3) Nsp1, Nsp2, Nsp7, Nsp13, Orf3a, Orf6, Orf7a and Orf7b inhibit TNF-α-induced NF-κB activation. Further analysis by IF, immunoblotting and RT-qPCR demonstrated that Nsp6 and Orf8 specifically inhibit the type I IFN response, and Orf6 suppresses TNF-α-induced NF-κB activation.

Nsp1 inhibits the ISG56.1, ISRE and NF-κB reporter expression. Given the fact that SARS-CoV-2 Nsp1 blocks host mRNA translation through interaction with 40S ribosomal subunit, the inhibition observed in the reporter assays might be due to the shutdown of host cell translation (Schubert et al., 2020). A previous study also demonstrated that Nsp1 inhibits the response to type I IFNs by suppressing IFN-α-induced STAT1 and STAT2 phosphorylation (Xia et al., 2020). However, whether Nsp1 directly inhibits the IRF3 and NF-κB pathways is uncertain.

Coronavirus Nsp6 forms a complex with Nsp3 and Nsp4 to induce double-membrane vesicles that play an essential role in viral RNA replication and transcription (Santerre et al., 2020). Nsp6 is a membrane protein with six transmembrane domains and a conserved C terminus as shown for the murine coronavirus MHV (Baliji et al., 2009). A previous study demonstrated that Nsp6 from multiple coronaviruses restricts autophagosome expansion, which may favor virus infection by suppressing autophagosome-mediated degradation of viral components (Cottam et al., 2014). In this study, ectopic expression of SARS-CoV-2 Nsp6 suppressed the ISRE-driven reporter expression, suggesting that Nsp6 is a novel inhibitor of the response to type I IFN. Subsequent analysis of IFN-α-induced STAT2 translocation and ISGs expression indicated the inhibition is through suppressing STAT2 translocation into the nucleus. Consistent with this Xia et al. (2020) also reported that Nsp6 inhibits the type I IFN response; yet the reduction of STAT phosphorylation observed is inconsistent with our results showing no impact of Nsp6 on the phosphorylation level of STAT1. Additionally, they showed inhibition of a reporter gene assay under control of the IFN-β promoter *via* interaction with TBK1, indicating a potential inhibitory activity targeting IRF3 or NF-κB. However, Nsp6 was not shown to inhibit either ISG56.1 or NF-κB reporter expression here, suggesting the mechanism by which Nsp6 inhibits expression from an IFN-β reporter is unclear.

SARS-CoV-2 variants lacking Orf8 expression were reported worldwide, providing valuable information about SARS-CoV-2 virology and pathogenesis (Young et al., 2020; Hill et al., 2022). Virus infection and replication analysis *in vitro* also demonstrate a non-essential role of Orf8 in SARS-CoV-2 (Gamage et al., 2020). The 382-nucleotide deletion in Orf8 in the variant isolated from Singapore seems to be associated with milder disease outcome, suggesting that Orf8 may contribute to virulence (Young et al., 2020). Consistent with the clinical observation, Zhang et al. (2021a) found that Orf8 of SARS-CoV-2 downregulates MHC-I molecules *in vitro*. Further studies using reporter gene assays showed that Orf8 inhibits SeV-induced IFN-β, ISRE and NF-κB responsive reporter expression, suggesting Orf8 regulates innate immune responses (Li et al., 2020). Consistent with this, our studies found that Orf8 suppresses the response to type I IFN, but not SeV-stimulated IRF3 or TNF-α-induced NF-κB activation. Our studies also show that Orf8 retains STAT1 in the cytoplasm after IFN-γ stimulation, indicating it likely also inhibits the response to type II IFN. Future studies using transduced cells lines expressing Orf8 would enable investigation of its function in the type II IFN response. Therefore, our studies and others demonstrated that Orf8 is a negative regulator of the response to type I IFN, but its role in SARS-CoV-2 infection *in vivo* is still unclear.

Orf6 of SARS-CoV is a potent IFN antagonist and has 69% aa identity with its SARS-CoV-2 counterpart (Frieman et al., 2007; Kopecky-Bromberg et al., 2007; Yuen et al., 2020). Several studies using IFN-β reporter assays identified Orf6 as a strong inhibitor of IFN-β production, which suggests that Orf6 interferes with IRF3, AP-1 or NF-κB pathways (Lei et al., 2020; Miorin et al., 2020; Xia et al., 2020; Yuen et al., 2020). Our study investigated the inhibitory activities of SARS-CoV-2 Orf6 on different immune pathways. ISG56.1 reporter expression, which is driven by IRF3 activation, is inhibited by Orf6. Consistent with previous reports (Miorin et al., 2020; Xia et al., 2020; Yuen et al., 2020; Miyamoto et al., 2022), we found that Orf6 inhibits the production of type I IFN and the response to it. IF analysis showed that Orf6 blocks the translocation of STAT2 and STAT1 into the nucleus following induction by type I and II IFN, respectively. Subsequent analysis of IFN-α-induced ISG expression demonstrated that SARS-CoV-2 Orf6 is a potent inhibitor of IFN-induced signal transduction. A recent study showed that Orf6 interacts with STAT1 *via* its C terminus to promote SARS-CoV-2 replicon reporter expression (Miyamoto et al., 2022). However, the interaction was not observed in a previous SARS-CoV-2 interactome study (Gordon et al., 2020), indicating the bioactivities and interaction partners of Orf6 require further investigation. In addition, the NF-κB reporter assay showed that Orf6 negatively regulates the TNF-α-stimulated NF-κB activation and IF analysis showed Orf6 inhibited p65 translocation into the nucleus. The following co-precipitation assay found that Orf6 does not interact with p65, indicating the inhibition of p65 translocation is mediated by a third binding partner. Previous studies found that the NF-κB subunits p50 and p65 interact with KPNA2 (Fagerlund et al., 2005), an interaction partner of SARS-CoV-2 Orf6 (Miorin et al., 2020), suggesting that Orf6 might interfere with KPNA2-mediated translocation of p65.

Coronaviruses express multiple inhibitors of innate immune pathways to escape from antiviral action and thus promote virus replication. In this study, SARS-CoV-2 Nsp6, Orf6 and Orf8 were identified as potent inhibitors of innate immune signaling pathways. Nsp6 inhibits the response to type I IFN by suppressing STAT2 translocation into nucleus. Orf8 also inhibits the response to IFN-α, but the mechanism is unclear. Orf6 is a potent inhibitor of IRF3 activation, as reported here and elsewhere. Orf6 also inhibits the response to type I and II IFN by blocking the nuclear translocation of STAT2 and STAT1. Multiple SARS-COV-2 proteins were shown to inhibit TNF-α-stimulated NF-κB activation. Further analysis showed that Orf6 suppresses this pathway by tethering p65 in the cytoplasm, thus preventing its translocation. In summary, data presented in this study identified new SARS-CoV-2 inhibitors of innate immune pathways. It will be important to address the contribution of these inhibitors to the virology and pathogenesis of SARS-CoV-2.

## Data availability statement

The raw data used to create the figures shown in this articles are available at Figshare doi: 10.6084/m9.figshare.21354099.

## Author contributions

YL and GS: conceptualization, methodology, and supervision. YL, HM, and GS: investigation and writing—original draft preparation. P-HW: resources. YL, HM, P-HW, and GS: writing—review and editing. GS: project administration and funding acquisition. All authors contributed to the article and approved the submitted version.

## Funding

This work was supported by the Wellcome Trust. GS was a Wellcome Trust Principal Research Fellow (grant #090315). YL was supported by the Wellcome Trust and the Isaac Newton Trust, Cambridge, grant 21.23(e). P-HW was supported by the Key Research and Development Program of Shandong Province (2020CXGC011305 to P-HW) and the National Key R&D Program of China (2021YFC2701203 to P-HW). HM was supported by the Master’s Fund of Selwyn College, University of Cambridge.

## Conflict of interest

The authors declare that the research was conducted in the absence of any commercial or financial relationships that could be construed as a potential conflict of interest.

## Publisher’s note

All claims expressed in this article are solely those of the authors and do not necessarily represent those of their affiliated organizations, or those of the publisher, the editors and the reviewers. Any product that may be evaluated in this article, or claim that may be made by its manufacturer, is not guaranteed or endorsed by the publisher.

## Supplementary material

SUPPLEMENTARY FIGURE S1 The expression of SARS-CoV-2 non-structure proteins in the reporter gene assay. Aliquots of cell lysates collected from Figure 1A were analyzed by immuno-blotting with anti-FLAG and anti-alpha-tubulin antibodies. The expression of viral proteins are indicated by red arrows. The positions of molecular mass markers in kDa are indicated on the right.
