# Supplementary material for: Manipulation of innate immune signaling pathways by SARS-CoV-2 non-structural proteins
Source: Front Microbiol. 2022 Nov 21;13:1027015. doi: 10.3389/fmicb.2022.1027015 (PMC9720297; doi:10.3389/fmicb.2022.1027015)
Supplement: SUPPLEMENTARY FIGURE S1 — The expression of SARS-CoV-2 non-structure proteins in the reporter gene assay. Aliquots of cell lysates collected from Figure 1A were analyzed by immuno-blotting with anti-FLAG and anti-alpha-tubulin antibodies. The expression of viral proteins are indicated by red arrows. The positions of molecular mass markers in kDa are indicated on the right. [file Table_1.DOCX]

| Target Gene | Sequence |
| --- | --- |
| *NFKBIA_*FWD | CTCCGAGACTTTCGAGGAAAT |
| *NFKBIA*_REV | GCCATTGTAGTTGGTAGCCTT |
| *CCL2*_FWD | CAGCCAGATGCAATCAATGCC |
| *CCL2*_REV | TGGAATCCTGAACCCACTTCT |
| *CXCL10*_FWD | GTGGCATTCAAGGAGTACCTC |
| *CXCL10*_REV | GCCTTCGATTCTGGATTCAGACA |
| *GAPDH*_FWD | ACCCAGAAGACTGTGGATGG |
| *GAPDH*_REV | TTCTAGACGGCAGGTCAGGT |

**Table S1**
